# Supplementary material for: Head and body structure infants’ visual experiences during mobile, naturalistic play
Source: PLoS One. 2020 Nov 10;15(11):e0242009. doi: 10.1371/journal.pone.0242009 (PMC7654772; doi:10.1371/journal.pone.0242009)
Supplement: Attachment — Submitted filename: Response to Reviewers.pdf [file pone.0242009.s001.pdf]

Response to Reviews

Below, we address the editor and reviewer comments point-by-point (our responses follow in italics).

Editor's Comments

1. Please ensure that your manuscript meets PLOS ONE's style requirements, including those for file naming.
We used PLOS ONE's LaTeX template to prepare the manuscript, and we have ensured that the manuscript meets PLOS ONE's style requirements.

2. Please provide additional details regarding participant consent. In the ethics statement in the Methods and online submission information, please ensure that you have specified whether consent was informed.
The ethics statement has been added in the method section.

3. We note that you have stated that you will provide repository information for your data at acceptance. Should your manuscript be accepted for publication, we will hold it until you provide the relevant accession numbers or DOIs necessary to access your data.
Noted. We will share our data on a Databrary repository and provide the DOI if the paper is accepted.

4. We note that Figure [1] includes an image of a patient / participant in the study. Please amend the methods section and ethics statement of the manuscript to explicitly state that the patient/participant has provided consent for publication: "The individual in this manuscript has given written informed consent (as outlined in PLOS consent form) to publish these case details".
The ethics statement has been added to the method section.

Reviewer 1's Comments

1. Head-centered field of view
How was the center of the scene camera image aligned to the center of the field of view of the infant's right eye (i.e., how was the head-centered field of view defined)? I believe that calibration in the eye tracker is done so as to match the position of the pupil in the eye-camera image and the location of the object in the scene camera image. The eye tracker can track the gaze accurately even if the orientation of scene camera is not perfectly aligned to the center of the actual field of view of the right eye. Slight changes in the orientation of the scene camera could result in different "center" in the scene camera image. In other words, although the eye tracker is able to pick up the location of gaze (green cross in Fig. 4) accurately, the center of the scene camera image and resulting distance between the image center and the green cross may not be so accurate.
We appreciate the comment. We agree that the field of view was not perfectly head-centered as the scene camera was positioned slightly over the right eye. However, given that the distance between the right eye and head midline is very small, the scene could be used as an approximation of the head-centered field of view. We are also aware of the concern about whether the scene camera is in fact oriented towards the center. We added to the method section to detail the calibration procedure that the original study used to ensure that the scene camera was positioned correctly.

2. Non-looking episodes
By definition, the toys in non-looking episodes were what randomly got in infant's field of view. They were not something that infant's visual system was oriented to. As such, I'm not sure whether and how the analysis of the data of "non-looking" episode could deepen our understanding the nature of infants' visual experiences during naturalistic tasks. I have difficulty in interpreting the results of the analysis of what infants were not looking at, and the comparison between looking and non-looking episodes.

Although we agree that what is in infant's field of view when not looking may be unintentional, we disagree with calling it random. Our aim is to show that infants' head and body movements shape their visual experiences, which may then shape their opportunities for learning about (and looking at) different stimuli in the environment. This is analogous to studies of auditory experiences—audio recorders count how much language infants are exposed to without knowing how much infants attend to. We added a paragraph at the end of the current study section to address this concern.

3. What does the result imply that far toys are less centered compared to close toys not only in looking episodes but also in "non-looking" episodes when infants are in prone (Fig. 6A)?
It implies that infants while prone have a more constrained field of view in general compared to in sitting and upright positions. Far toys are more likely to become out of view (as they are located at the top of the field of view) for infants while prone during both looking and non-looking episodes. It may be because lifting the head to look at distant objects in prone is effortful. We added a clearer interpretation of this result to the discussion.

4. Why did toy locations in the field of view become more spread from prone to sitting and from sitting to upright during "non-looking" episodes, but not during looking episodes (Fig. 6B)? What underlies the increased spread in the gaze toward closely located toys while infants are prone in looking episodes (Fig 6B), which was the exact opposite to the tendency found in "non-looking" episode just mentioned above?
We think that infants in sitting and upright positions have a relatively higher point of view than prone and have visual access to a larger area of the environment. That's why the spread becomes greater from prone to sitting and from sitting to upright—a larger viewing area allows a larger spread of object positions. This trend is not shown during looking episodes because infants actively center the object in view, reducing the spread of positions while looking.

Regarding why the spread of close toys while prone is larger than in other positions during looking episodes, we suggest that infants have more difficulty stabilizing the field of view while prone. Tilting the head up and down is effortful while prone. As a result, infants are less likely to keep the toys at consistent locations in the field of view when looking. However, we believe this warrants future testing. We added these interpretations of the results to the discussion.

5. The effect of distance
The authors used the distance in the 2D image of the scene camera to measure how centered and variable toy locations in infants' field of view. Close toy occupies the greater area of the scene camera image (Fig 3) than far toy does (Fig 4). I wonder if there are greater chances for eye gaze (green cross) to fall near the ROI when a toy is closely located and takes up the scene camera image compared to the situation where the toy is located far. If the actual distance between the object and the infant is available, visual angle instead of distance on 2D image might be a better measure, as it may compensate for such an effect.
We appreciate the comment. Yes, close toys are more likely to be looked at than far toys in the real world. In the study, we used the center of the toy (not the edges of the toy) to calculate the distance from the toy to the center of field of view. Thus, the visual size of a toy in the field of view would not affect the calculation of centering and spread. We acknowledge that the actual distance between the object and the infant would be a better measure. But unfortunately, that information is not available.

Reviewer 2's Comments

Introduction

The authors state, "Although it is a useful technique to examine some aspects of infants' selective attention, it precludes measuring the motor aspects of attention and how they shape infants' visual experiences of objects in daily life." Researchers who use SET to examine infants' visual attention may classify eye movement as "motor movements." It would be useful for the authors to specify that they are referring to gross motor movements. This would strengthen their argument about the role of body position on visual attention in infancy.

We appreciate this suggestion. The phrases have been revised.

Method

Under procedure, is there a publication that can be cited to elaborate on the calibration procedure?
The references of the calibration procedure have been added.

Results

Please include units for Tables 1 and 3.
The units have been added.

Discussion

I think that an additional limitation or perhaps future direction could be pairing visual attention with the probability of reaching toward particular objects. This would offer information about visually guided reaching and prospective control with upper extremity tasks in infancy.
Thank you for the suggestion. This point has been added to the discussion.
